# EFFECTS OF ASSISTIVE TENNIS SHOES ON GROUND REACTION FORCE FOR PATIENTS WITH PERIPHERAL ARTERY DISEASE DURING WALKING

**DOI:** 10.1093/geroni/igac059.2538

**Published:** 2022-12-20

**Authors:** Nathaniel Evans, Hafizur Rahman, Iraklis Pipinos, Jason Johanning, Mahdi Hassan, Sara Myers

**Affiliations:** University of Nebraska Omaha, Omaha, Nebraska, United States; University of Nebraska at Omaha, Omaha, Nebraska, United States; University of Nebraska Medical Center, Omaha, Nebraska, United States; University of Nebraska Medical Center, Omaha, Nebraska, United States; University of Nebraska at Omaha, Omaha, Nebraska, United States; University of Nebraska at Omaha, Omaha, Nebraska, United States

## Abstract

Peripheral artery disease (PAD) is caused due to buildup of atherosclerotic plaques, typically in the leg arteries, preventing adequate blood circulation and ultimately claudication. A previous study showed that the vertical ground reaction force (VGRF) curve is significantly flatter in claudicating patients, resulting in a lower and less fluctuant center of mass when ambulating. Patients with PAD also demonstrate significantly decreased propulsion forces in the anterior– posterior direction. Assistive tennis shoes (carbon fiber: CF, and spring-loaded: SL) can potentially assist push-off by substituting for muscle forces using energy stored in a carbon fiber plate or metal spring within the shoe. This study aims to examine how the CF and SL shoes impact walking performance in patients with PAD. A total of ten patients with PAD performed a progressive treadmill test using a pressure-instrumented treadmill for each shoe type: i) standard shoes, ii) CF shoes, and iii) SL shoes. We calculated the peak VGRF for three subjects to date as an average of ten stance phases for the beginning of the walking condition (pain free condition). Preliminary results from three subjects showed that patients with PAD generated a greater peak VGRF wearing CF and SL shoes compared to normal shoes during the heel contact (normal: 0.97±0.10BW, CF: 1.03±0.08BW, and SL: 1.09±0.10BW) and push-off (normal: 0.97±0.06BW, CF: 0.99±0.04BW, and SL: 1.03±0.05BW). In future, we will calculate the VGRF for the remaining patients in pain free and pain conditions and conduct statistical analysis to identify significant differences among shoe types.

